# Coronary Artery Bypass Surgery in a Patient With an Occluded Abdominal Aorta Supported by Microaxial Left Ventricular Assist Device

**DOI:** 10.1016/j.atssr.2025.09.031

**Published:** 2025-10-30

**Authors:** Gina DeFelice, Niti Dalal, Jamil Borgi, Aabha Divya

**Affiliations:** 1 Division of Cardiothoracic Surgery, Department of Surgery, Tulane School of Medicine, New Orleans, Louisiana; 2 Department of Epidemiology, Tulane School of Public Health and Tropical Medicine, New Orleans, Louisiana

## Abstract

Coronary artery disease with severe left ventricular dysfunction presents perioperative challenges during bypass surgery when vascular access is limited. Traditional mechanical circulatory support options, such as intraaortic balloon pumps, may be unsuitable in patients with peripheral vascular disease. We present the case of a 63-year-old man with severe coronary artery disease with an occluded infrarenal aorta precluding standard device placement. An Impella 5.5 (Abiomed) was successfully implanted perioperatively via the left axillary artery. Although typically used in high-risk percutaneous coronary interventions and cardiogenic shock, this case highlights this device’s role in supporting low ejection fraction coronary artery bypass grafting.

Patients with severe multivessel coronary artery disease and reduced left ventricular ejection fraction undergoing coronary artery bypass grafting (CABG) are at elevated risk for perioperative cardiogenic shock and low cardiac output syndrome, frequently necessitating high-dose inotropic support to maintain hemodynamic stability.^1^ The intraaortic balloon pump (IABP), when placed perioperatively, has been shown to improve surgical outcomes in this population.^2^ However, vascular limitations such as peripheral artery disease and injuries preclude the use of IABP.^3^ In this case, IABP was not feasible due to an occluded infrarenal aorta and prior right brachial plexus injury, which precluded right axillary access. Instead, the Impella 5.5 (Abiomed) was placed via the left axillary artery perioperatively, providing effective hemodynamic support and reducing postoperative inotropic needs.

A 63-year-old male patient with a history of non-insulin-dependent diabetes mellitus, hyperlipidemia, and hypertension presented to the emergency department with non–ST-segment elevation myocardial infarction, post percutaneous coronary intervention (PCI; Ramus intermedius 2.5 x 16mm Synergy XD DES and OM 2-2.5 x 16mm Synergy XD DES), with chest pain, dyspnea, and diaphoresis. His medical history was further complicated by severe peripheral vascular disease and chronic contracture of the right upper extremity.

Preoperative coronary angiography confirmed severe multivessel disease involving the left main trifurcation, consistent with the indication for surgical revascularization. Transthoracic echocardiography demonstrated a reduced left ventricular ejection fraction of 20% with regional wall motion abnormalities. Computed tomography angiogram confirmed that the abdominal aorta was completely occluded, just distal to the origins of the renal arteries, with celiac and superior mesenteric artery stenosis. Extensive collaterals between the internal mammary and intercostal arteries and the inferior epigastric arteries provide flow to the external and internal iliac arteries bilaterally. The right axillary artery was found to be severely stenotic, measuring 0.6 cm in diameter (Figure).

**Figure:**
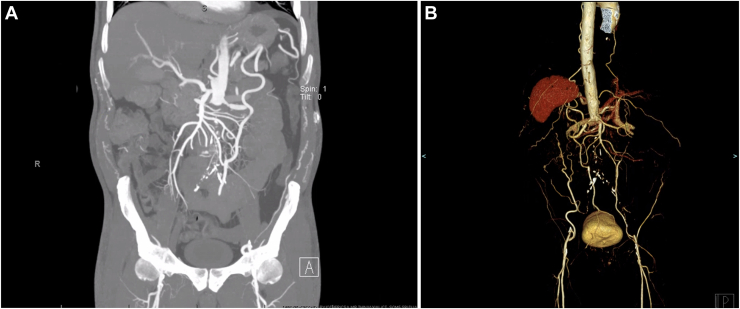
Coronal computed tomography angiogram (A) and 3-dimensional volume rendered image (B) of the abdomen demonstrating complete occlusion of the infrarenal abdominal aorta distal to the origin of the renal arteries. Extensive collateral vessels from the internal thoracic and intercostal arteries are visualized supplying the iliac circulation bilaterally.

CABG was planned for the multivessel coronary artery disease. However, femoral access for IABP was not feasible due to bilateral infrarenal aortic occlusion. Due to prior trauma and chronic contracture from a motor vehicle accident, the right axillary artery was unsuitable. Attempts to wean off cardiopulmonary bypass resulted in increasing inotropic support and unstable hemodynamics. Hence, the patient was put on Impella 5.5 via the left axillary artery. Postoperatively, the patient remained hemodynamically stable, requiring minimal inotropic support (norepinephrine at 0.05 μg/kg/min and dobutamine at 5 μg/kg/min) with a device flow rate of 3.5 L/min.

The patient was extubated on postoperative day (POD) 1. The device support was completely weaned off on POD 4. After device removal, a repeat transthoracic echocardiography demonstrated an improvement in ejection fraction. The patient was transferred out of the intensive care unit and discharged home subsequently in stable condition.

Per institutional policy, institutional review board review was not required for single-patient case reports. Written informed consent was obtained from the patient for publication of this case report and any accompanying images.

## Comment

Patients undergoing CABG with severe left ventricle dysfunction are at increased risk for postoperative low cardiac output syndrome and cardiogenic shock.^4^ Traditionally, IABPs have been utilized to optimize hemodynamics in these patients.^3^^,^^5^ However, peripheral vascular disease and prior vascular injuries may limit vascular access options for IABP insertion.

The Impella 5.5 has been widely utilized in cases of high-risk PCI and cardiogenic shock.^6^ Our case report demonstrates that perioperative Impella 5.5 placement in a high-risk CABG candidate improved hemodynamic stability and reduced the need for high-dose inotropic and pressor support postoperatively. Additionally, it provides an ambulatory capacity, which is particularly advantageous for postoperative recovery.

Patients with peripheral vascular disease present unique challenges in mechanical circulatory support device placement. The presence of an occluded abdominal aorta, as in the case presented, further complicates access options.^5^ The right axillary artery is typically preferred, but prior trauma or vascular complications precluded its use in our case. In these cases, careful preoperative imaging is necessary. We placed the Impella 5.5 via the left axillary artery. This ensured adequate ventricular unloading and circulatory support. The device provides continuous ventricular unloading, reducing myocardial oxygen demand and improving perfusion, facilitating myocardial recovery.^4^ Several studies have suggested that prophylactic placement of Impella 5.5 in high-risk cardiac surgery patients can mitigate perioperative hemodynamic instability, leading to improved outcomes.^6^

The patient was successfully weaned off inotropic support by POD 5, confirming myocardial recovery. This aligns with findings from Ramzy and associates,^7^ which reported higher survival rates in patients receiving Impella 5.5 for surgical support compared to historical controls.

A study by Ranganath and colleagues^4^ demonstrated that temporary mechanical circulatory support in high-risk CABG patients resulted in early extubation, reduced inotropic requirements, and favorable hemodynamics postoperatively. Similarly, this patient achieved early mobilization, transitioned to step-down care by POD 6, and was discharged home by POD 9.

Goldstein and coworkers^5^ emphasized that Impella 5.5 provides effective ventricular unloading and reduces dependence on inotropes. Furthermore, Thalji and associates^8^ identified that patients with severe left ventricle dysfunction undergoing CABG are at significantly higher risk for postoperative mortality, suggesting that prophylactic Impella placement may improve outcomes in this subgroup. Extracorporeal membrane oxygenation provides alternative circulatory support; it is associated with higher bleeding risks and limited mobility. Impella 5.5 offers superior left ventricular unloading with lower complication rates, making it a more favorable choice for temporary perioperative support.

In conclusion, although Impella has been primarily utilized in high-risk PCI and cardiogenic shock, this case demonstrates its utility in supporting low ejection fraction CABG. In patients with peripheral vascular disease and contraindications to IABP or femoral access, axillary artery device placement provides an effective alternative for perioperative hemodynamic support.

This case reinforces the growing role of Impella 5.5 in complex cardiac surgery, particularly in patients who may not be candidates for conventional circulatory support devices. Future studies are needed to define patient selection criteria and optimal management strategies for integrating Impella 5.5 into high-risk cardiac surgical protocols.
